# Prevalence, Impact, and Screening Methods of Sarcopenia in Japanese Patients With Parkinson’s Disease: A Prospective Cross-Sectional Study

**DOI:** 10.7759/cureus.65316

**Published:** 2024-07-24

**Authors:** Keishu Murakami, Jinsoo Koh, Shuhei Ogami, Yohei Aoki, Kohei Hori, Seiji Emori, Takuya Matsumoto, Junko Taruya, Shoko Yorozu, Mayumi Sakata, Yoshiaki Nakayama, Katsuichi Miyamoto, Hidefumi Ito

**Affiliations:** 1 Department of Neurology, Wakayama Medical University, Wakayama, JPN; 2 Department of Neurology, National Hospital Organization Wakayama Hospital, Wakayama, JPN; 3 Department of Occupational Therapy, Kansai University of Health Sciences, Osaka, JPN

**Keywords:** awgs 2019 consensus, calf circumference, screening method, sarcopenia, parkinson' s disease

## Abstract

Introduction: Sarcopenia is a skeletal muscle disease manifesting as low muscle mass and impaired muscle function. It has been reported that sarcopenia correlates with a low quality of life (QOL) and an increased risk of falls in patients with Parkinson's disease (PD). Nevertheless, few studies have investigated the prevalence, impact, and screening methods of sarcopenia in Japanese patients with PD.

Methods: Sarcopenia was diagnosed based on the Asian Working Group for Sarcopenia 2019 consensus. We compared demographic characteristics, severity of PD, levodopa equivalent daily dose, QOL, fatigue, impulsive and compulsive behaviors, body mass index (BMI), calf circumference, skeletal muscle mass index (SMI), handgrip strength, a 4-meter gait speed, a five-time sit-to-stand test (FTSST), short physical performance battery, and SARC-F questionnaire scores between sarcopenia and non-sarcopenia groups. Furthermore, to investigate the best tool for screening sarcopenia in PD, the sensitivity and specificity of calf circumference, handgrip strength, FTSST, and SARC-F questionnaire were compared.

Results: The prevalence of sarcopenia in PD was 31.9% (15/47). The sarcopenia group showed significantly higher age (77.3 ± 5.12 versus 70.3 ± 8.17, p = 0.0042), lower BMI (19.3 ± 2.99 versus 23.3 ± 3.18, p = 0.0002), higher rate of decreased calf circumference (86.6% versus 34.3%, p = 0.0013) and SMI (100% versus 6.25%, p < 0.0001), and worse FTSST (15.5 ± 5.57 versus 12.0 ± 4.12, p = 0.0219). The other parameters were not significantly different. Among screening tools, calf circumference had the highest sensitivity (86%) and specificity (65%). All screening tools had higher sensitivity and specificity in men than in women. The SARC-F questionnaire was not useful in distinguishing sarcopenia but was significantly correlated with the Movement Disorder Society-sponsored revision of the Unified Parkinson's Disease Rating Scale Part 3 (r = 0.41, p = 0.0037) and the 39-item Parkinson’s Disease QOL Scale (r = 0.71, p < 0.0001).

Conclusion: This study investigated the characteristics of PD patients with sarcopenia in Japan. Calf circumference was found to be the most useful tool for screening sarcopenia in PD. Handgrip strength and FTSST also showed high sensitivities, particularly in men. Conversely, the SARC-F questionnaire is not suitable for diagnosing sarcopenia in PD.

## Introduction

Sarcopenia is a skeletal muscle disease characterized by low muscle mass and impaired muscle function [1]. Sarcopenia has arisen as a significant global public health concern because of its increasing prevalence [2] and high rate of adverse outcomes, such as falls, disability, poor quality of life (QOL), institutionalization, hospitalization, and death [3]. Similarly, in patients with Parkinson's disease (PD), sarcopenia was found to be correlated with a low QOL and an increased risk of falls [4]. Given that exercise therapy improves sarcopenia in patients with PD, it is crucial to diagnose and intervene in the early stages of sarcopenia [5]. The review of epidemiology studies from Asian countries discovered that the prevalence of sarcopenia was affected by various factors such as country, sex, and clinical conditions [6]. Thus, it is important to elucidate the prevalence of sarcopenia in Japanese patients with PD. Nevertheless, few studies have been conducted on sarcopenia in Japanese patients with PD.

The Asian Working Group for Sarcopenia (AWGS) 2019 consensus recommends diagnosing sarcopenia based on decreases in skeletal muscle mass, muscle strength, and physical performance [7]. The AWGS 2019 consensus recommends the use of calf circumference or a SARC-F questionnaire for case-finding, and handgrip strength or a five-time sit-to-stand test (FTSST) for assessment in primary healthcare [7]. Previous reports have shown that the SARC-F questionnaire is not suitable for screening for sarcopenia in patients with PD [8] but is a good predictor of disabilities in PD [9]. However, the utility of calf circumference, handgrip strength, and the FTSST for screening sarcopenia based on the AWGS 2019 consensus in Japanese patients with PD has not yet been investigated.

The aim of this study was to examine the prevalence and impact of sarcopenia in Japanese patients with PD. In addition, we investigated the utility of calf circumference, handgrip strength, the FTSST, and the SARC-F questionnaire in the screening of sarcopenia in patients with PD.

## Materials and methods

Study design and participants

**Figure 1:**
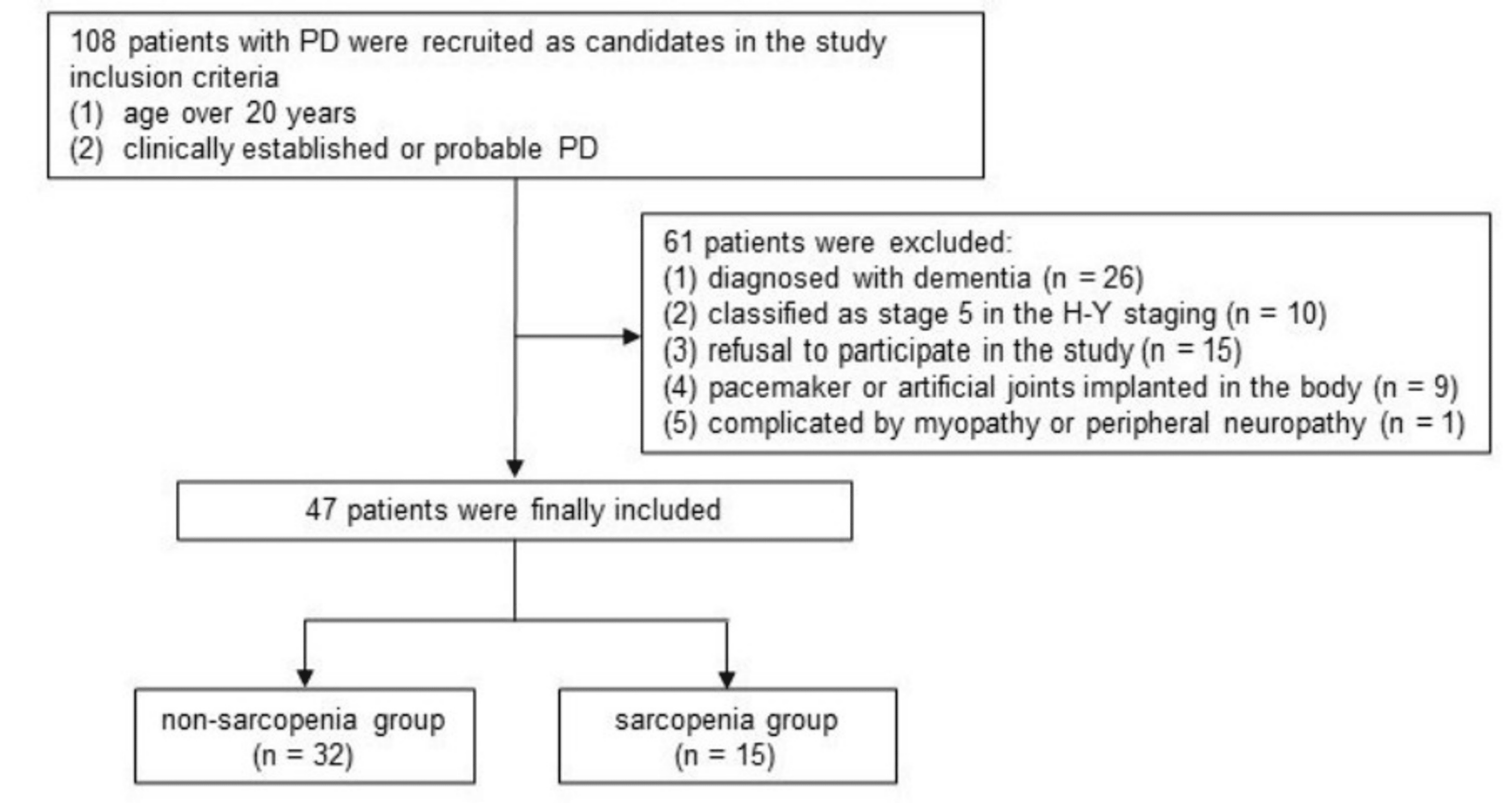
Flowchart of patient selection In total, 108 patients with PD were recruited for this study. After excluding 61 patients, 47 were enrolled and divided into the sarcopenia (n = 15) and non-sarcopenia (n = 32) groups. PD: Parkinson's disease; H-Y: Hoehn and Yahr

This prospective cross-sectional study was conducted in a Japanese population. A total of 108 patients with PD were recruited as candidates in the National Hospital Organization Wakayama Hospital, Wakayama, Japan from October 1, 2023, to March 31, 2024. Patients were enrolled based on the following inclusion criteria: age over 20 years and diagnosis of clinically established or probable PD according to the Movement Disorder Society Clinical Diagnostic Criteria for PD [10]. Patients meeting the following criteria were excluded: diagnosis of dementia, stage 5 in the Hoehn and Yahr (H-Y) staging [11], refusal to participate in this study, implantation of pacemaker or artificial joints in the body, and complication of myopathy or peripheral neuropathy. Based on these criteria, 47 patients with PD were finally included in this study (Figure 1). All the participants provided written informed consent to participate. This study was approved by the Local Ethics Committee of the National Hospital Organization Wakayama Hospital (approval number: 05-4; approval date: September 20, 2023). All procedures involving human participants were performed in accordance with the National Ethical Guidelines for Biomedical and Health Research and the Declaration of Helsinki.

Data collection

Demographic information was collected from the medical records, including age, sex, comorbidities (hypertension, dyslipidemia, osteoporosis, and type 2 diabetes), disease duration of PD, and levodopa equivalent daily dose (LEDD) [12]. The educational backgrounds of the participants were assessed based on the number of years of education completed. The H-Y stages and Movement Disorder Society-sponsored revision of the Unified Parkinson's Disease Rating Scale (MDS-UPDRS) Part 3 and Part 4 scores [13] were assessed by the clinical neurology specialists, Keishu Murakami and Shuhei Ogami. Participants were categorized into being mild (H-Y ≦ 2) or moderate-severe (H-Y ≧ 3). Dyskinesia and motor fluctuations were recorded based on MDS-UPDRS Part 4 scores. Cognitive function was assessed by Mini-Mental State Examination. QOL, fatigue, and impulsive and compulsive behaviors (ICBs) were evaluated using the 39-item Parkinson’s Disease Quality of Life Scale (PDQ-39) [14], the 16-item Parkinson Fatigue Scale (PFS-16) [15], and the Questionnaire for Impulsive-Compulsive Disorders in Parkinson's Disease-Rating Scale (QUIP-RS) [16], respectively. We previously validated the Japanese version of PDQ-39 [17] and QUIP-RS [18] by the translation and back-translation method and obtained confirmation from the original authors. The validation of the Japanese version of PFS-16 was performed for the assessment of fatigue in patients with PD [19,20]. The SARC-F questionnaire is a brief and straightforward screening tool for sarcopenia, for which a total score of ≧4 is indicative of sarcopenia and associated with unfavorable clinical outcomes, as previously reported [21]. Tanaka et al. carried out translation and adaptation of the original scale to Japanese with permission from the corresponding author [22].

Diagnosis of sarcopenia

Sarcopenia was diagnosed in accordance with the AWGS 2019 consensus [7]. The AWGS 2019 consensus defines sarcopenia and severe sarcopenia as separate categories. However, in this study, sarcopenia and severe sarcopenia were treated as a single group, termed a sarcopenia group.

Anthropometric measurements and evaluation of body composition

Height and weight were measured using a DP-5200 stadiometer and digital floor scale (Yamato Scale, Akashi, Japan). The patient’s body mass index (BMI) was calculated by dividing weight by the square of height. Calf circumference was measured on the widest part of the left calf using a millimeter-scale measuring tape (Abbott Japan, Tokyo, Japan). Appendicular skeletal muscle mass was quantified using InBody S10 (InBody Japan, Tokyo, Japan) between 9:00 a.m. and 10:00 a.m. before measurements of muscle strength and physical performance were conducted. The test was performed after excretion and under fasting conditions. Appendicular skeletal muscle mass was adjusted for the square of the height to define the skeletal muscle mass index (SMI). Decreased SMI was defined as <7.0 kg/m^2^ in men and <5.7 kg/m^2^ in women [7].

Muscle strength

Handgrip strength was measured using a TKK 5401 digital dynamometer (Takei Scientific Instruments, Tokyo, Japan). Measurements were conducted with patients in the standing position, with the elbow in the extended position. Handgrip strength was quantified twice for each hand, and the average value of a total of four measurements was used as the patient's handgrip strength. Decreased muscle strength was defined as < 28.0 kg in men and < 18.0 kg in women [7].

Physical performance

Physical performance was evaluated using the 4-meter gait test, FTSST, and short physical performance battery (SPPB). In the 4-meter gait test, gait speed was calculated as the time taken to walk 4 meters at a normal pace from the start of movement without deceleration, and low gait speed was defined as <1.0 meter/second [7]. In the FTSST, the participants were instructed to stand up from a seated position and sit down five times as quickly as possible from a chair with a height of 40 cm. During this task, participants were required to keep their arms folded across their chests. The interval between the start and the participant’s arrival at the chair on the fifth repetition was recorded using a manual stopwatch [4]. The threshold for low physical performance, as determined by the FTSST, was ≧12 seconds [7]. The SPPB is based on three tasks: standing balance, walking speed, and chair stand test [23]. The original SPPB employed an 8-feet gait test to evaluate walking speed, whereas the present study utilized a 4-meter gait test, as previously described [24]. The results of each subtest were rescaled according to predefined cut-off points, with scores ranging from 0 (worst performance) to 4 (best performance). The total score was calculated by summing the scores of each subtest. The cutoff point for low physical performance on the SPPB was ≦9 [7].

Statistical analysis

Statistical analyses were performed using JMP Pro 14.1.0. (SAS Institute Inc., Cary, USA). Data are presented in accordance with the results of the Shapiro-Wilk test. Data exhibiting a normal distribution are presented as means with standard deviations, while non-normally distributed data are presented as the median (interquartile range). Categorical parameters are expressed as frequencies and percentages. Statistical analysis of the differences between the study groups with continuous variables was conducted using either the Student's t-test or the Mann-Whitney U-test. Categorical variables were analyzed using Fisher's exact test. Receiver operating characteristic curve analysis was conducted to examine the diagnostic value of calf circumference, handgrip strength, FTSST, and SARC-F questionnaire for the screening of sarcopenia. Sensitivity and specificity were calculated using the cutoff values adopted in the AWGS 2019 consensus. Three separate analyses of the FTSST were performed in men only, women only, and both men and women. Finally, scatter plot analysis was performed and Pearson's correlation coefficients were calculated to examine whether or not the SARC-F questionnaire scores were correlated with the MDS-UPDRS Part 3 or PDQ-39 scores. Statistical significance was set at p < 0.05.

## Results

Demographic characteristics and clinical data of patients with PD

Table 1 shows the demographic characteristics and clinical data of 47 patients with PD. The mean age of women was higher than that of men, but the difference was not statistically significant. Women had fewer years of education, higher rates of hypertension and osteoporosis, and worse cognitive function than men. Conversely, there were no statistically significant differences between men and women in disease duration, MDS-UPDRS Part 3 and Part 4 scores, LEDD, QOL, fatigue, or ICBs. Among them, 15 (31.9%) patients with PD, including 3 men and 12 women, were diagnosed with sarcopenia or severe sarcopenia in accordance with the AWGS 2019 consensus. Sex differences were not correlated with any of the following variables: BMI, the SARC-F questionnaire scores, calf circumference, SMI, handgrip strength, physical performance tests, and the prevalence of sarcopenia.

**Table 1:** Demographic characteristics and clinical data of patients with PD Measurement data are shown as mean ± standard deviations in a normal distribution in accordance with the results of the Shapiro-Wilk test, while presented as the median (interquartile range) in a non-normal distribution. The categorical parameters were written with the use of frequencies and percentages. a: Student's t-test; b: Fisher's exact test; c: Mann-Whitney U-test PD: Parkinson's disease; MMSE: Mini-Mental State Examination; MDS-UPDRS: Movement Disorder Society-sponsored revision of the Unified Parkinson's Disease Rating Scale; H-Y: Hoehn and Yahr; LEDD: levodopa equivalent daily dose; PDQ-39 SI: 39-item Parkinson’s Disease Quality of Life Scale summary index; PFS-16: 16-item Parkinson Fatigue Scale; QUIP-RS: Questionnaire for Impulsive-Compulsive Disorders in Parkinson's Disease-Rating Scale; BMI: body mass index; SMI: skeletal muscle mass index; FTSST: five-time sit-to-stand test; SPPB: short physical performance battery

Variable	Total (n = 47)	Men (n = 17)	Women (n = 30)	p-value
Age, years old	72.5 ± 7.98	69.7 ± 8.85	74.2 ± 7.09	0.0611^a^
Education ≦ 12 years, n (%)	31 (65.9)	7 (41.1)	24 (80.0)	0.0108^b^
Comorbidities				
Hypertension, n (%)	27 (57.4)	6 (35.2)	21 (70.0)	0.0320^b^
Dyslipidemia, n (%)	18 (38.2)	5 (29.4)	13 (43.3)	0.5332^b^
Osteoporosis, n (%)	16 (34.0)	1 (5.88)	15 (50.0)	0.0031^b^
Type 2 diabetes, n (%)	8 (17.0)	4 (23.5)	4 (13.3)	0.4350^b^
MMSE	28 (25, 30)	29 (27, 30)	26.5 (24.7, 29)	0.0151^c^
Disease duration, years	6 (3, 8)	5 (3, 10.5)	6 (4, 8)	0.6801^c^
MDS-UPDRS Part 3	18.3 ± 8.54	17.7 ± 7.31	18.6 ± 9.27	0.7323^a^
MDS-UPDRS Part 4	0 (0, 5)	0 (0, 6)	0 (0, 5)	0.8810^c^
Motor fluctuations, n (%)	16 (34.0)	5 (29.4)	11 (36.6)	0.7526^b^
Dyskinesia, n (%)	14 (29.7)	6 (35.2)	8 (26.6)	0.7409^b^
H-Y stage ≧ 3, n (%)	10 (21.2)	2 (11.7)	8 (26.6)	0.2894^b^
LEDD, mg/day	450 (325, 700)	450 (250, 627)	475 (385, 755)	0.1868^c^
PDQ-39 SI	25.2 ± 13.9	21.9 ± 8.98	27.0 ± 15.8	0.1658^a^
PFS-16 ≧ 3.3, n (%)	19 (40.4)	4 (23.5)	15 (50.0)	0.1220^b^
QUIP-RS	0 (0, 10)	3 (0, 13.5)	0 (0, 7.25)	0.1137^c^
BMI, kg/m^2^	22.0 ± 3.61	22.2 ± 3.37	21.9 ± 3.80	0.7881^a^
SARC-F ≧ 4, n (%)	22 (46.8)	5 (29.4)	17 (56.6)	0.1273^b^
Calf circumference < cutoff, n (%)	25 (53.1)	6 (35.2)	18 (60.0)	0.1351^b^
SMI < cutoff, n (%)	17 (36.1)	4 (23.5)	13 (43.3)	0.2181^b^
Handgrip strength < cutoff, n (%)	27 (57.4)	8 (47.0)	19 (63.3)	0.3617^b^
Gait speed, meters/second	1.09 ± 0.37	1.21 ± 0.42	1.02 ± 0.33	0.0984^a^
FTSST, seconds	12.4 (9.64, 16.1)	11.6 (10.3, 15.7)	12.7 (8.76, 16.6)	0.8403^c^
SPPB	21 (44.6)	11 (8, 12)	9.5 (7, 12)	0.2693^c^
Sarcopenia, n (%)	15 (31.9)	3 (17.6)	12 (40.0)	0.1926^b^

Differences in clinical characteristics of PD between the sarcopenia and non-sarcopenia groups

Table 2 shows the demographic characteristics and clinical features of the patients with PD in the sarcopenia and non-sarcopenia groups. We found statistically significant differences in age and BMI between the sarcopenia and non-sarcopenia groups. However, sarcopenia was not correlated with sex differences, educational background, comorbidities, disease duration, MDS-UPDRS Part 3 and Part 4 scores, motor fluctuations, dyskinesia, H-Y stages, LEDD, QOL, fatigue, or ICBs. The SARC-F questionnaire scores were equal between the two groups, whereas calf circumference was associated with sarcopenia. The number of patients with decreased handgrip strength was greater in the sarcopenia group; however, the difference did not reach statistical significance (p = 0.0563). In this study, three types of physical performance tests were conducted; the 4-meter gait test, FTSST, and SPPB. The results of all three tests tended to be worse in the sarcopenia group; however, only the FTSST showed a statistically significant difference between the two groups (p = 0.0219).

**Table 2:** Difference of demographic characteristics and clinical data between sarcopenia and non-sarcopenia group Measurement data are shown as mean ± standard deviations in a normal distribution in accordance with the results of the Shapiro-Wilk test, while presented as the median (interquartile range) in a non-normal distribution. The categorical parameters were written with the use of frequencies and percentages. a: Student's t-test; b: Fisher's exact test; c: Mann-Whitney U-test MMSE: Mini-Mental State Examination; MDS-UPDRS: Movement Disorder Society-sponsored revision of the Unified Parkinson's Disease Rating Scale; H-Y: Hoehn and Yahr; LEDD: levodopa equivalent daily dose; PDQ-39 SI: 39-item Parkinson’s Disease Quality of Life Scale summary index; PFS-16: 16-item Parkinson Fatigue Scale; QUIP-RS: Questionnaire for Impulsive-Compulsive Disorders in Parkinson's Disease-Rating Scale; BMI: body mass index; SMI: skeletal muscle mass index; FTSST: five-time sit-to-stand test; SPPB: short physical performance battery

Variable	Non-sarcopenia group (n = 32)	Sarcopenia group (n = 15)	p-value
Age, years old	70.3 ± 8.17	77.3 ± 5.12	0.0042^a^
Female, n (%)	18 (56.2)	12 (80.0)	0.1926^b^
Education ≦ 12 years, n (%)	19 (59.3)	12 (80.0)	0.2024^b^
Comorbidities			
Hypertension, n (%)	19 (59.3)	8 (53.3)	0.7583^b^
Dyslipidemia, n (%)	13 (40.6)	5 (33.3)	0.7526^b^
Osteoporosis, n (%)	9 (28.1)	7 (46.6)	0.3224^b^
Type 2 diabetes, n (%)	5 (15.6)	3 (20.0)	0.6974^b^
MMSE	28 (26, 30)	26 (25, 29)	0.3359^c^
Disease duration, years	5.5 (3, 10.3)	6 (4, 7)	1.0000^c^
MDS-UPDRS Part 3	18.0 ± 8.82	19.0 ± 8.18	0.6947^a^
MDS-UPDRS Part 4	0 (0, 5.75)	0 (0, 5)	0.7872^c^
Motor fluctuations, n (%)	10 (31.2)	6 (40.0)	0.7422^b^
Dyskinesia, n (%)	11 (34.3)	3 (20.0)	0.4957^b^
H-Y stage ≧ 3, n (%)	6 (18.7)	4 (26.6)	0.7042^b^
LEDD, mg/day	413 (300, 765)	500 (400, 666)	0.5369^c^
PDQ-39 SI	25.8 ± 12.6	24.0 ± 16.7	0.7162^a^
PFS-16 ≧ 3.3, n (%)	12 (37.5)	7 (46.6)	0.7507^b^
QUIP-RS	2 (0, 12)	0 (0, 4)	0.1413^c^
BMI, kg/m^2^	23.3 ± 3.18	19.3 ± 2.99	0.0002^a^
SARC-F ≧ 4, n (%)	15 (46.8)	7 (46.6)	1.0000^b^
Calf circumference < cutoff, n (%)	11 (34.3)	13 (86.6)	0.0013^b^
SMI < cutoff, n (%)	2 (6.25)	15 (100)	< 0.0001^b^
Handgrip strength < cutoff, n (%)	15 (46.8)	12 (80.0)	0.0563^b^
Gait speed, meters/second	1.12 ± 0.39	1.03 ± 0.33	0.4311^a^
FTSST, seconds	12.0 ± 4.12	15.5 ± 5.57	0.0219^a^
SPPB	11 (8, 12)	8 (7, 10)	0.0542^c^

Diagnostic utility of calf circumference, handgrip strength, FTSST, and SARC-F questionnaire for sarcopenia screening in patients with PD based on the AWGS 2019 consensus

**Figure 2:**
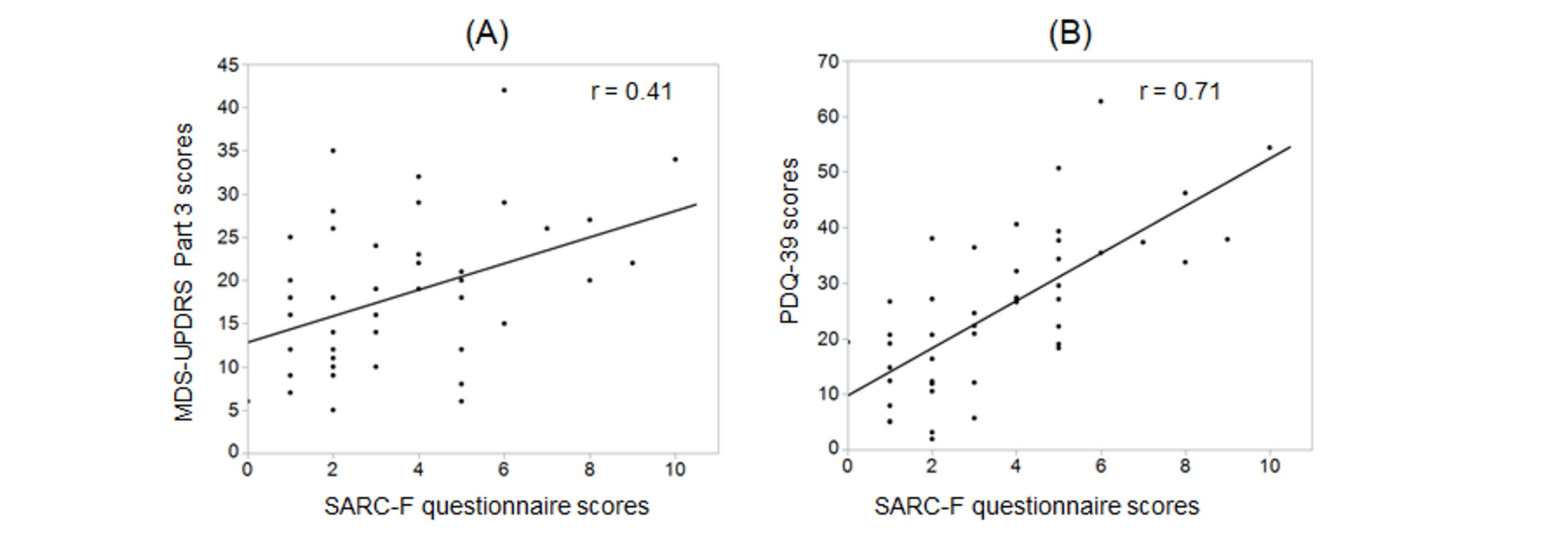
Correlation of the SARC-F questionnaire scores with severities or QOL of PD Scatter plot analysis showed that SARC-F scores correlated with (A) the MDS-UPDRS Part 3 (r = 0.41, p = 0.0037) and (B) PDQ-39 scores (r = 0.71, p < 0.0001). QOL: quality of life; PD: Parkinson's disease; MDS-UPDRS: Movement Disorder Society-sponsored revision of the Unified Parkinson's Disease Rating Scale; PDQ-39: 39-item Parkinson’s Disease Quality of Life Scale; r: Pearson's correlation coefficients

Receiver operating characteristic curve analysis was performed to assess the potential of calf circumference, handgrip strength, FTSST, and SARC-F questionnaire for screening sarcopenia in patients with PD (Table 3). In men, the sensitivity of calf circumference, handgrip strength, and FTSST was 1.00, indicating that all three tests were useful in the exclusion of sarcopenia. The most specific test for the detection of sarcopenia in men was calf circumference. In contrast, the sensitivity and specificity of calf circumference, handgrip strength, and FTSST were all lower in women than in men. Calf circumference was the most sensitive and specific of the three tests in women. The sensitivity of the SARC-F questionnaire was much lower than those of calf circumference, handgrip strength, and FTSST. The SARC-F questionnaire scores correlated with the MDS-UPDRS Part 3 (Figure 2A) and PDQ-39 scores (Figure 2B).

**Table 3:** Comparison of the methods for the screening of sarcopenia in patients with PD Receiver operating characteristic curve analysis was conducted to examine AUC, sensitivity, and specificity of calf circumference, handgrip strength, FTSST, and SARC-F questionnaire. PD: Parkinson's disease; AUC: area under the curve; NA: not available; FTSST: five-time sit-to-stand test

	AUC	Sensitivity (%)	Specificity (%)
Calf circumference			
Men < 34 cm	0.94	100	78
Women < 33 cm	0.82	83	55
Men + women	NA	86	65
Handgrip strength			
Men < 28 kg	0.78	100	64
Women < 18 kg	0.58	75	44
Men + women	NA	80	53
FTSST ≧ 12 seconds			
Men	0.84	100	71
Women	0.64	83	50
Men + women	0.71	86	59
SARC-F ≧ 4	0.51	46	53

## Discussion

In this study, we demonstrated that 31.2% of patients with PD had sarcopenia. This finding aligns with a prior systematic review, which reported a pooled prevalence of sarcopenia in patients with PD at 29% (95% confidence interval: 18-40%) [25]. In contrast, the prevalence of sarcopenia in Japanese community-dwelling older adults (age ≧ 65) ranges from 4.9% to 13.2% [26-28]. Thus, sarcopenia appears to be more prevalent in patients with PD than in non-PD elderly adults. Moreover, our study might underestimate the prevalence of sarcopenia because we excluded PD patients with dementia and those in H-Y stage 5, both conditions where sarcopenia is more common [29].

Several hypotheses have been proposed to explain the high prevalence of sarcopenia in patients with PD [25]. First, sarcopenia and PD may share common neuroinflammatory pathways, with elevated interleukin-6 levels observed in patients with both conditions [30]. Second, reductions in gray matter volume and a decreased default mode network play significant roles in the pathophysiology of both PD and sarcopenia [31]. Finally, neurogenic sarcopenia, defined as the simultaneous occurrence of sarcopenia and a pathologically low number of motor units, was observed in patients with PD, suggesting that a decrease in the number of motor neurons could induce sarcopenia, although the detailed mechanisms have remained unclear [32]. Therefore, while there is a possibility that sarcopenia is prevalent in PD, further research is needed to confirm these hypotheses and to elucidate its underlying causes.

Identifying optimized screening tools for sarcopenia in patients with PD is essential. The AWGS 2014 consensus recommended sarcopenia screening for the community-dwelling elderly and people with specific clinical conditions [6]. In the AWGS 2019 consensus, the SARC-F questionnaire, calf circumference, or the combination of the SARC-F questionnaire with calf circumference (i.e., SARC-Calf) are recommended for case finding [7]. However, in this study, the SARC-F questionnaire failed to differentiate sarcopenia in patients with PD. While the SARC-F questionnaire has shown a sensitivity of 17.9% and specificity of 93.7% for screening sarcopenia in community-dwelling older adults [33], its sensitivity and specificity in PD patients were 33.3% and 47.3%, respectively [34]. Furthermore, Ozer et al. indicated that the SARC-F questionnaire was a better predictor of PD severity and disability than sarcopenia [9]. Similarly, in this study, the SARC-F questionnaire scores correlated with the MDS-UPDRS Part 3 and PDQ-39 scores. Hence, it is inferred that in patients with PD, the SARC-F questionnaire is associated with PD severity and may not be suitable for case finding.

Conversely, calf circumference was shown to be an effective screening tool for sarcopenia in this study, consistent with previous research in Brazil [34]. Notably, the utility of calf circumference differed between men and women, with lower sensitivity and specificity observed in women. We speculate that the discrepancy between calf circumference and skeletal muscle mass observed in women is attributable to the greater subcutaneous fat deposits. Additionally, handgrip strength and the FTSST were more useful for men than for women. Therefore, it is necessary to develop better screening tools for sarcopenia in female patients with PD than calf circumference, handgrip strength, and the FTSST.

The impact of sarcopenia on PD patients remains poorly understood. Drey et al. reported a significant relationship between the UPDRS Part 3 score and early sarcopenia, suggesting a common pathway between the two conditions [35]. Lima et al. claimed that PD patients with sarcopenia have a higher risk of falling and a lower QOL than those without sarcopenia [4]. Recently, sarcopenia has been linked to non-motor symptoms of PD, including sleep quality and fatigue severity [36]. However, in this study, there were no significant differences in PD severity, LEDD, QOL, fatigue, or ICBs between the sarcopenia and non-sarcopenia groups. These discrepancies with previous studies may stem from methodological differences. A previous study used the SARC-F questionnaire instead of the SMI to define sarcopenia, leading to potential inaccuracies in diagnosing sarcopenia in PD patients [4]. Additionally, variations in diagnostic criteria for sarcopenia (AWGS 2019 consensus versus the European Working Group on Sarcopenia in Older People 2), PD symptom evaluation (UPDRS versus MDS-UPDRS), and assessments of fatigue (fatigue severity scale versus PFS-16) and QOL (PDQ-8 versus PDQ-39) could explain the observed discrepancies. Furthermore, the limited sample size in this study may have prevented the detection of statistically significant differences. Given the scarcity of studies on sarcopenia in Japanese PD patients based on the AWGS 2019 consensus, further research with larger sample sizes is warranted.

This study has several limitations. First, as mentioned above, the limited sample size may have led to an underestimation of statistical differences. Second, the disproportion of sex differences might affect the results. Third, bioelectrical impedance analysis (BIA) used for measuring SMI may be less accurate than dual-energy X-ray absorptiometry because BIA is affected by body water content [37]. Fourth, selection biases might exist. Finally, methodological differences prevent direct comparison with previous reports. The AWGS 2019 consensus recommends a 6-meter walk [7], and the original SPPB [23] includes an 8-foot walk, while this study used a 4-meter walk test for comparison with our previous studies. Although walking tests with distances less than 10 meters do not affect gait speed in healthy subjects [38], the impact of walking distance cannot be ruled out in PD patients.

## Conclusions

This study investigated the prevalence, impact, and diagnostic methods used to screen Japanese patients with PD for sarcopenia. The prevalence of sarcopenia in this cohort was approximately 30%, which is consistent with previous studies. Sarcopenia showed no correlation with PD characteristics in this study. Calf circumference, handgrip strength, and the FTSST were useful tools for excluding sarcopenia in men, whereas the sensitivity and specificity of the three tests were lower in women than in men. This result indicates that the measurement of SMI is more important for the diagnosis of sarcopenia in women than in men. The SARC-F questionnaire correlated with the severity and QOL of patients with PD and was not useful for the exclusion of sarcopenia. However, this study has several limitations. Thus, further studies are required to develop more straightforward and comprehensive methods for assessing sarcopenia in patients with PD.
